# Multiple Percutaneous Drainage of a Giant Pyonephrosis Caused by Urolithiasis: A Case Report

**DOI:** 10.7759/cureus.39684

**Published:** 2023-05-30

**Authors:** Dragos Puia, Stefan Gheorghinca, Catalin Pricop

**Affiliations:** 1 Urology, “Grigore T. Popa” University of Medicine and Pharmacy, Iasi, ROU; 2 Urology, Neamt County Emergency Hospital, Piatra Neamt, ROU

**Keywords:** solitary functional kidney, giant hydronephrosis, urinary tract infection, nephrostomy, kidney stones

## Abstract

The prevalence of kidney stones continues to rise in modern times. Undiagnosed and/or mistreated, it can result in suppurative kidney damage and, in rare instances, death from systemic infection. We present the case of a 40-year-old woman who presented to the county hospital for sleight left lumbar pain, fever, and pyuria for about two weeks. Ultrasound and CT scan revealed a giant hydronephrosis with no visible parenchyma, secondary to a stone in the pelvic-ureteral junction. Although a nephrostomy stent was placed, 48 hours later the purulent content was not evacuated completely. She was referred to a tertiary center, where two more nephrostomy tubes were placed to completely evacuate approximately 3 L of purulent urine. Three weeks later, after the inflammation parameters normalized, a nephrectomy was performed with good outcomes.

A pyonephrosis urologic emergency can develop into septic shock, demanding rapid medical attention to prevent potentially fatal outcomes. In some circumstances, percutaneous draining of a purulent collection may not be sufficient to remove the whole purulent mass. Before nephrectomy, all collections must be removed with further percutaneous procedures.

## Introduction

Kidney stones are nowadays a disease with an increased prevalence. Unrecognized and/or untreated, it can lead to suppurative destruction of the kidney and, in some cases, death of the patient secondary to systemic infection [1]. According to Rabii et al., kidney stones could be caused by pyelo-ureteral junction syndrome, prostate cancer, and retroperitoneal tumors, but the main etiology is urolithiasis in up to 70% of cases [2]. Typical clinical manifestations are high fever, chills, and/or flank pain. Although the final treatment is a nephrectomy, in patients with signs of urosepsis, the first step is emergency drainage to release the pressure in the upper urinary system and reduce the risk of septic shock. We present the case of a patient who required three percutaneous nephrostomies for proper drainage before definitive treatment.

## Case presentation

A 40-year-old woman with no medical history presented to the county hospital for sleight left lumbar pain, fever (38.6 °C), and pyuria for approximately two weeks.

Diagnostic assessment

**Figure 1:**
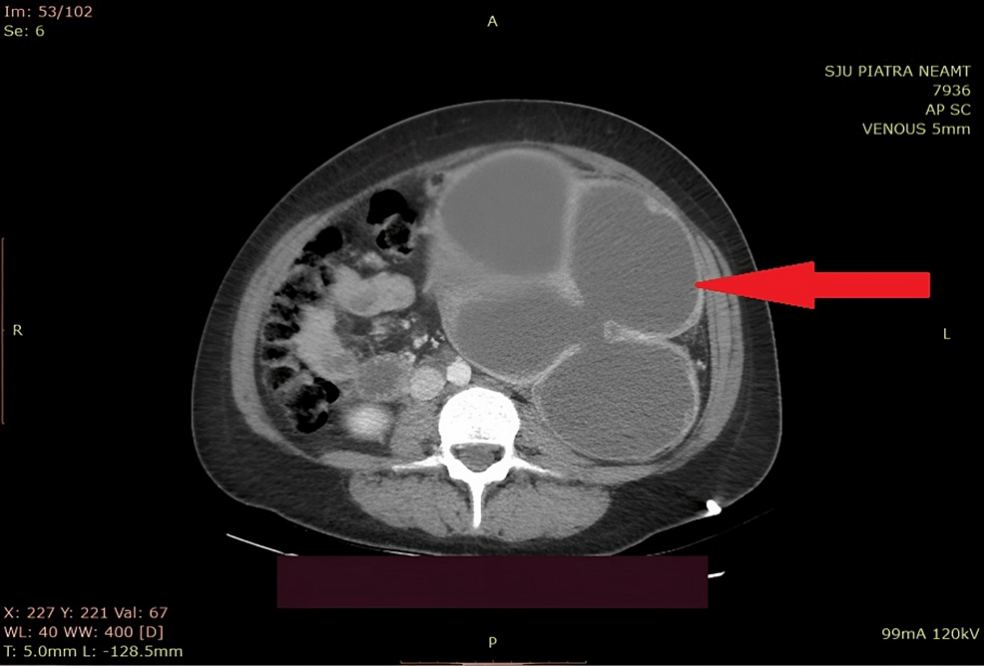
The CT scan showing the left kidney as a huge cystic mass

Laboratory tests showed normal serum creatinine and an inflammatory reaction, hyperleukocytosis 15.36x109/L, C-reactive protein 234 mg/L, international normalized ratio (INR) 1.39, and prothrombin time 23.7 seconds. Ultrasound revealed a giant left cystic mass with no visible renal parenchyma. The contrast-enhanced CT scan showed the left kidney as a substantial cystic mass with multiple septa secondary to a 23 mm x 18 mm kidney stone in the ureteropelvic junction. The kidney showed no signs of functionality, and the parenchyma was extremely thin (Figure 1).

Therapeutic interventions

**Figure 2:**
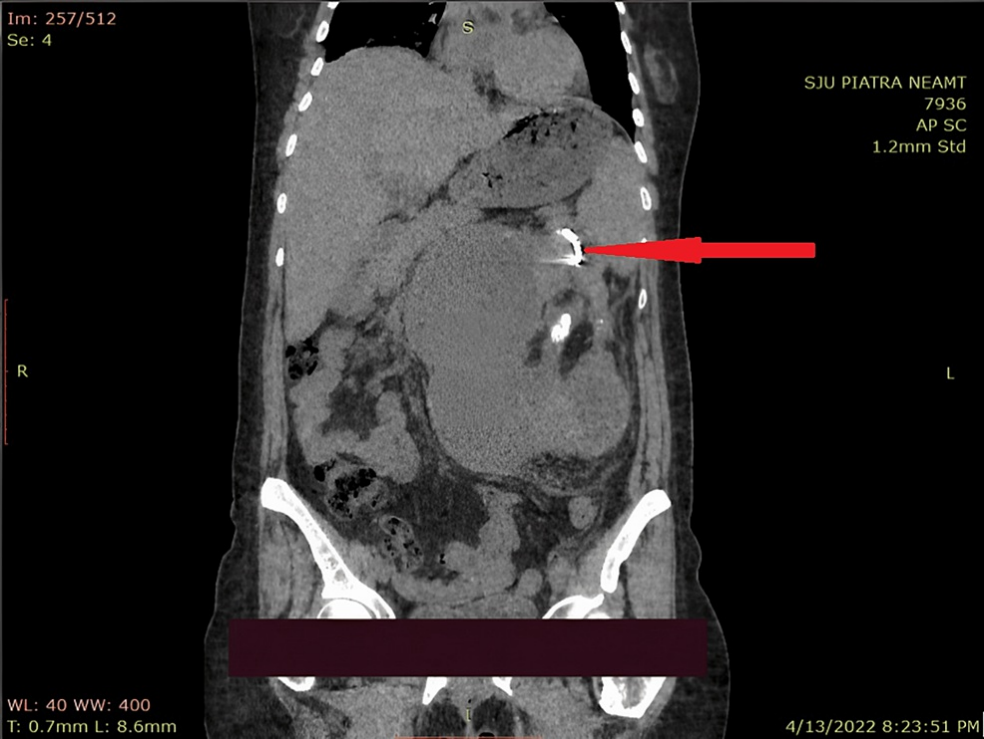
CT scan showing the stent in the collecting system but with the incomplete evacuation of the infected urine

The patient received antibiotic therapy with meropenem 1 g every eight hours, and a percutaneous nephrostomy was performed. After the drainage of over 2 L of purulent liquid, the culture revealed three different microbial strains, multidrug-sensitive *Escherichia coli* being the predominant one. Forty-eight hours later, the imagistic evaluation showed the stent in the collecting system. Still, the evacuation of the infected urine was incomplete (Figure 2). The patient was referred to a tertiary center to complete the treatment. The blood analysis in the tertiary center showed hemoglobin of 9.1 g/dl, hematocrit of 26.3%, serum creatinine 0.47 mg/dl, WBC 36.9x109/L, platelet count 565 x 109/L, procalcitonin 19.27 ng/ml, and C-reactive protein 154 mg/L. Two more nephrostomy tubes were placed to evacuate almost 3 L of purulent urine. Two days later, the blood analysis revealed a WBC of 9.25x109/L. Three weeks after, a simple total nephrectomy was performed with good outcomes.

## Discussion

Giant pyonephrosis is an uncommon condition. It represents a suppurative infection of the upper urinary system that varies from uninfected hydronephrosis to when pus is present. The primary etiology is ureteral obstruction. According to Rabii et al., kidney stones are the most common obstructive cause of pyonephrosis. In contrast, according to Hassen et al., ureteropelvic junction syndrome is the most frequent cause of giant hydronephrosis [2,3]. Having an infectious substrate, the clinical manifestations are represented by flank pain with fever and chills, although in 15% of cases, it could be an incidental finding [4].

Being readily available, renal ultrasound is the first imagistic method for evaluating individuals with renal inflammatory disease. According to Tamburriniet al., ultrasonography has a sensitivity of 90% for distinguishing hydronephrosis from pyonephrosis and a specificity of 97% for diagnosing pyonephrosis [4]. When performing renal sonography, if the hydronephrosis shows hyperechoic debris, the diagnosis of pyonephrosis is very suggestive. A CT scan has a higher diagnostic sensitivity, although recent studies have demonstrated that patients with pyonephrosis had higher HU levels in the dilated collecting system than in the case of simple non-complicated hydronephrosis, CT evaluation has its limitations in distinguishing simple hydronephrosis from pyonephrosis by fluid attenuation measurements [5]. The CT scan can reveal the underlying etiology, like urolithiasis and tumor pathology. Nowadays, intravenous urogram (IVU) is only of historical interest because, unlike CT, it does not provide information related to the adjacent organs or the vascularization of the kidney.

The lack of a history of urinary catheters or other urologic surgery could explain the positive urine culture with multidrug-sensitive* E.coli*. In our service, multidrug-resistant *Klebsiella spp*. is the most common encounter in positive urine culture [6]. Urosepsis is the most feared consequence of pyonephrosis, with fatality rates ranging from 20% to 42% [7]. Antibiotics are ineffective unless the infected urine is surgically drained [7]. Percutaneous nephrostomy and ureteral catheter insertion are the first-line surgical treatment options. Thus, according to Erol et al., percutaneous drainage should be the treatment of choice in a patient with inflammatory syndrome because it is an effective diagnostic and, at the same time, the therapeutic method [8]. According to our previous data, the intervention should preferably be performed within the first six hours after admission, regardless of the chosen method. These patients have the most favorable evolution, short hospitalization, and, last but not least, the lowest treatment costs [9].

If the contralateral kidney is normal, a simple nephrectomy may be the ideal option for a kidney that has lost most of its function [5]. Due to changes secondary to chronic inflammation, nephrectomy can lead to some surgical complications. According to Selviet et al., the most common intraoperative incidents in these patients are injuries to the colon (most usually on the left), duodenum, inferior vena cava, laceration of the inferior pole of the spleen, and unintentional opening of the pleural or peritoneal cavity. Postoperative colic, ileus, severe thrombophlebitis, and surgical wound suppuration are common postoperative sequelae [10].

In our patient, a total of 5 L of pus was evacuated through nephrostomies. This volume is alike to the one reported by El Mostapha et al. in a similar case, also secondary to a ureteral stone. Some authors described even greater volumes, of 7 L to 11 L [8,11]. In all these cases, the first step of treatment has been drainage through percutaneous nephrostomy followed by nephrectomy.

Although rupture of pyonephrosis is also very rare, Niang et al. recently reported a retroperitoneal rupture with extension up to the crural muscles, treated successfully by percutaneous nephrostomy followed by nephrectomy [12]. Intraperitoneal ruptures are also possible. Shiftiet al. reported the case of a 28-year-old male patient with peritonitis secondary to left-sided pyonephrosis, which besides nephrectomy, also required abdominal lavage with good recovery [13].

## Conclusions

In specific cases of pyonephrosis, one percutaneous drainage of the purulent collection is not enough to evacuate all of the purulent mass. Our case highlights the necessity to eliminate all collections with the help of other percutaneous interventions before nephrectomy.
